# Utility of Shear Wave Elastography for Diagnosing Chronic Autoimmune Thyroiditis

**DOI:** 10.1155/2015/164548

**Published:** 2015-07-16

**Authors:** Takahiro Fukuhara, Eriko Matsuda, Shoichiro Izawa, Kazunori Fujiwara, Hiroya Kitano

**Affiliations:** ^1^Department of Otolaryngology-Head and Neck Surgery, Tottori University Faculty of Medicine, 36-1 Nishi-cho, Yonago, Tottori 683-8504, Japan; ^2^Endocrinology and Metabolism, Department of Molecular Medicine and Therapeutics, Tottori University Faculty of Medicine, 36-1 Nishi-cho, Yonago, Tottori 683-8504, Japan

## Abstract

The aims of this study were to evaluate the utility of shear wave elastography (SWE) using acoustic radiation force impulse (ARFI) for diagnosing chronic autoimmune thyroiditis (CAT) and to verify the effect of fibrotic thyroid tissue on shear wave velocity (SWV). The subjects were 229 patients with 253 normal thyroid lobes (controls) and 150 CAT lobes. The SWV for CAT (2.47 ± 0.57 m/s) was significantly higher than that for controls (1.59 ± 0.41 m/s) (*P* < 0.001). The area under the receiver operating characteristics (ROC) curve for CAT was 0.899, and the SWV cut-off value was 1.96 m/s. The sensitivity, specificity, and diagnostic accuracy were 87.4%, 78.7%, and 85.1%, respectively. Levels of anti-thyroperoxidase antibodies and thyroid isthmus thickness were correlated with tissue stiffness in CAT. However, there was no correlation between levels of anti-thyroglobulin antibodies and tissue stiffness. Quantitative SWE is useful for diagnosing CAT, and it is possible that SWE can be used to evaluate the degree of fibrosis in patients with CAT.

## 1. Introduction

Shear wave elastography (SWE) measures the shear wave velocity (SWV) generated by the acoustic radiation force impulse (ARFI). SWV reflects tissue elasticity, which can be calculated using Young's modulus [1]. Thus, SWE can be used to evaluate tissue stiffness, both quantitatively and objectively. Some authors have described the utility of SWE for differentiating thyroid nodules from normal surrounding tissue [2–8]; however, since the factors that affect SWV in thyroid lesions are unclear, the appropriate use of SWE for this purpose remains to be determined.

Virtual touch tissue quantification (VTQ) was originally used to evaluate the extent of liver fibrosis; the technique is well established in this context [9, 10]. Since the same principles can be applied to the thyroid, we hypothesize that SWE would be useful for diagnosing chronic autoimmune thyroiditis (CAT), which causes fibrosis in the thyroid parenchyma. Since the ARFI is not usually impaired in superficial tissues such as the thyroid, we hypothesize that shear waves will be more stable in the thyroid than in the liver.

Conventional strain elastography measures the stiffness of the target tissue relative to that of the surrounding tissue; therefore, it can only estimate the tissue strain induced by compression [11–14]. Thus, it is difficult to estimate the stiffness of a diffuse lesion using strain imaging because the technology cannot really estimate minor differences in stiffness. However, SWE may be helpful in the evaluation of patients for CAT by determining the extent of fibrosis. The aims of the present study were to examine the diagnostic accuracy of SWE for CAT and to verify the correlation between fibrosis and SWV in the thyroid.

## 2. Patients and Methods

### 2.1. Patients

Study subjects included 229 individuals, including 253 normal thyroid lobes (controls) and 150 lobes with CAT, who underwent ultrasonography of the thyroid gland in the Department of Otolaryngology-Head and Neck Surgery of Tottori University. This study, which was performed between November 2011 and October 2014, was approved by the Ethics Committee and the Institutional Review Board of Tottori University. Informed consent was obtained from all study participants. The study was performed in accordance with the Declaration of Helsinki.

### 2.2. Inclusion and Exclusion Criteria

The normal thyroid group had normal ultrasound findings, as well as normal serum levels of thyroid-stimulating hormone, free triiodothyronine, and free thyroxine. A diagnosis of CAT was based on elevated levels of thyroglobulin antibodies, anti-thyroperoxidase antibodies, or both, as well as a diffuse hypoechoic pattern in the thyroid tissue on ultrasonography [15, 16]. The study population included patients receiving L-thyroxine therapy, patients not receiving any therapy, euthyroid patients, and patients with subclinical or overt hypothyroidism, because Ruchala et al. have demonstrated that thyroid stiffness is not affected by L-thyroxine treatment [17]. CAT patients with goiter, normal thyroid volume, or an atrophic thyroid were included.

Patients with Grave's disease, a history of radiation therapy to the neck, a history of using medications that may cause secondary CAT, and a history of neck surgery that has altered the blood flow within the remnant thyroid were excluded. Generally, the left lobe is smaller than the right lobe. When the left lobe was not thick enough to encompass the region of interest (ROI), it was excluded from the analysis.

### 2.3. Pathological Review

In patients who underwent surgery for another indication, such as carcinoma, and received a pathological diagnosis of CAT, the degree of fibrosis was evaluated pathologically. We divided the degree of fibrosis into three groups: no fibrosis, moderate fibrosis, and severe fibrosis. We used ImageJ software (version 1.48, Wayne Rasband, National Institutes of Health, USA) to calculate the degree of fibrosis. Severe fibrosis was defined as extended fibrosis in over 50% of the area of 5 mm^2^ microscope fields. Moderate fibrosis indicated findings intermediate between no fibrosis and severe fibrosis. The average SWV of each group was compared. We measured SWV in the contralateral lobe as well.

### 2.4. Measurement Procedure

**Figure 1:**
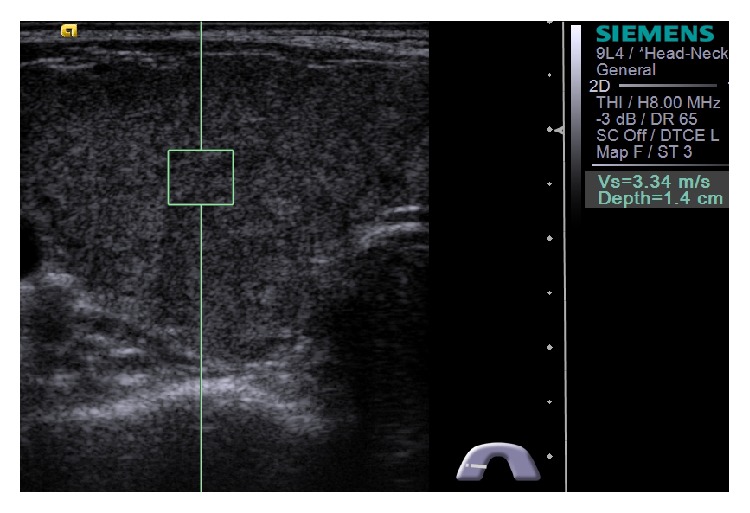
Image showing how measurements of shear wave velocity in thyroid were made. The 5 × 5 mm region of interest is entirely enclosed within the thyroid lobe.

Each lobe of the thyroid gland in control and CAT subjects was measured using a 9 MHz B-mode-ARFI combination linear transducer (ACUSON 2000, Siemens Medical Systems, Forchheim, Germany). SWE using ARFI was performed with the VTQ system (Siemens Medical Systems). Generally, VTQ was performed on B-mode ultrasonographic images as follows: first, a target region was identified within a ROI of 5 × 5 mm. An acoustic push pulse was then transmitted, with a shear wave generated within the target region. This shear wave was detected by sonographic detection pulses, and numerical SWV values were displayed. SWV was measured in each lobe of the thyroid glands by pressing the probe lightly against the neck in an axial direction. The ROI (5 × 5 mm) was completely circumscribed by the thyroid lesion (Figure 1). Sporea et al. reported that five separate measurements are sufficient to assess thyroid stiffness [18]; therefore, five measurements were performed in the same location, and the average was recorded for comparison.

### 2.5. Statistical Analysis

All data were expressed as means ± standard deviation (SD). Data were compared using the Mann-Whitney *U* test. Diagnostic performance was assessed using receiver operating characteristic (ROC) curves. This information was used to derive the optimal SWV cut-off value. The correlation between SWV and various factors associated with CAT, such as serum antibody titers and thyroid isthmus thickness, was evaluated using Spearman's correlation analysis. Statistical analysis was performed using SPSS software, version 22 (IBM Corporation, Armonk, NY).

## 3. Results

A total of 229 patients (90 males and 139 females; mean age, 58.2 years; range, 10–91 years) who were eligible according to the inclusion and exclusion criteria were examined. There were 253 normal thyroid lobes in 145 patients (83 males and 62 females; mean age, 57.5 years) and 150 CAT lobes in 84 patients (7 men and 77 women; mean age, 60.5 years).

**Figure 2:**
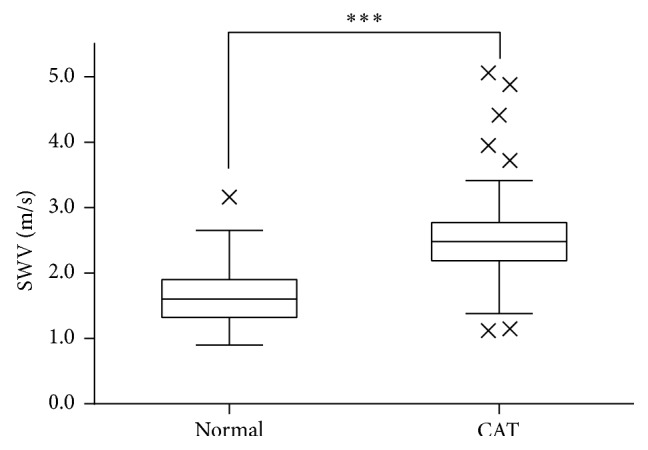
Box-and-whisker plots showing the shear wave velocity in each group. The symbol × indicates an outlier. ^*∗∗∗*^ *P* < 0.001.

The mean SWV for CAT was 2.47 ± 0.57 m/s, which was significantly higher than the mean SWV for normal controls (1.59 ± 0.41 m/s) (*P* < 0.001; Figure 2).

**Figure 3:**
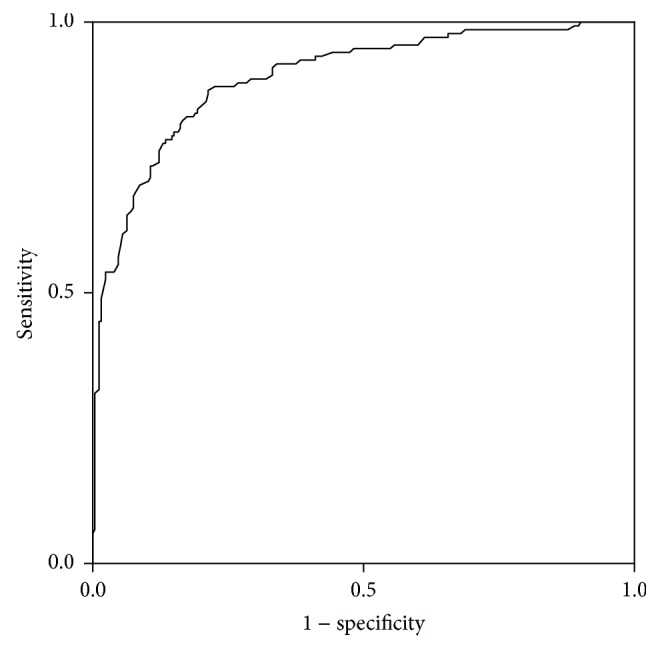
Receiver operating characteristic curve used to predict chronic autoimmune thyroiditis based on shear wave velocity.

The ROC curve for the thyroid lobes with CAT is shown in Figure 3. The area under the ROC curve (AUC) was 0.899, and the optimal SWV cut-off value was 1.96 m/s. The sensitivity, specificity, positive predictive value, negative predictive value, and diagnostic accuracy were 87.4%, 78.7%, 74.2%, 94.0%, and 85.1%, respectively.

A total of 17 CAT patients underwent resection. There were one patient with no fibrosis and SWV of 2.05 m/s, 11 patients with moderate fibrosis (mean SWV, 2.44 m/s), and three patients with severe fibrosis (mean SWV, 3.12 m/s).

**Figure 4:**
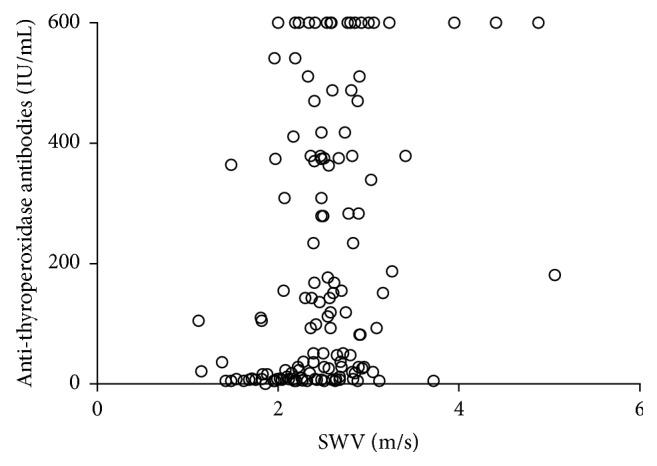
Correlation between shear wave velocity and serum titers of anti-thyroperoxidase antibodies.

**Figure 5:**
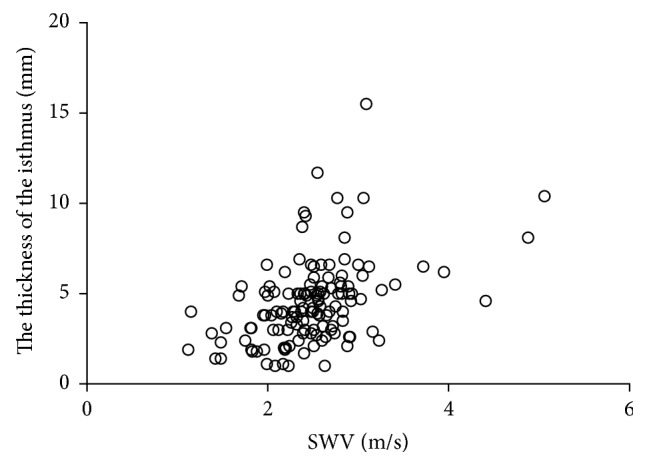
Correlation between shear wave velocity and thyroid isthmus thickness.

**Figure 6:**
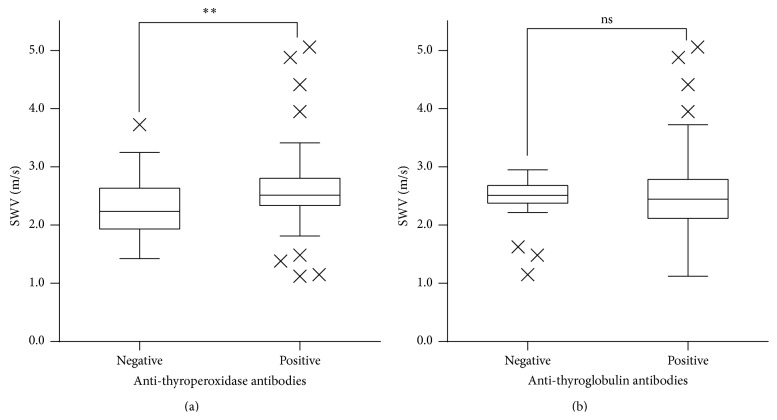
Box-and-whisker plots showing shear wave velocity by anti-thyroperoxidase and anti-thyroglobulin antibody status. (a) Note. ^*∗∗*^ *P* < 0.01.

There were 111 patients positive for anti-thyroperoxidase antibodies and 39 patients negative for anti-thyroperoxidase antibodies. There were 117 patients positive for anti-thyroglobulin antibodies and 33 patients negative for anti-thyroglobulin antibodies. We found a weak positive correlation between SWV and serum levels of anti-thyroperoxidase antibodies (Spearman's correlation coefficient, 0.311; Figure 4) and between SWV and thyroid isthmus thickness (Spearman's *ρ* = 0.441; Figure 5). There was no correlation between SWV and levels of serum anti-thyroglobulin antibodies (Spearman's *ρ* = 0.101). The mean SWV for CAT lobes with positive anti-thyroperoxidase antibodies was 2.56 ± 0.57 m/s, significantly higher than that for CAT lobes without detectable anti-thyroperoxidase antibodies (2.27 ± 0.51 m/s) (*P* = 0.002; Figure 6(a)). There were no significant differences in SWV between CAT lobes with anti-thyroglobulin antibodies (2.49 ± 0.61 m/s) and without anti-thyroglobulin antibodies (2.46 ± 0.39 m/s) (Figure 6(b)).

## 4. Discussion

SWV was significantly higher in thyroid lobes with CAT than normal thyroid tissue. The AUC was 0.899, indicating a high degree of diagnostic accuracy. Therefore, we conclude that quantitative SWE is useful for diagnosing CAT on ultrasonography. The finding of higher SWV in CAT than in control lobes shows that it is possible to use SWE to evaluate the fibrosis associated with CAT. The SWV in lobes with CAT correlated with both the thickness of the thyroid isthmus and the presence of anti-thyroperoxidase antibodies; both are parameters that increase with the duration of disease.

CAT-related fibrosis results in high SWV. The pathological features of CAT include interstitial infiltration by lymphocytes and a variable degree of fibrosis in the interstitium [15, 16, 19]. It is thought that fibrosis accelerates the propagation of the shear wave; therefore, the SWV in CAT should depend on the level of fibrosis in the thyroid tissue. This was corroborated by the results of pathological examination for resected specimens with CAT.

Conventional strain elastography measures the stiffness of the target tissue relative to that of the surrounding tissue; therefore, it can only estimate the tissue strain induced by compression [11–14]. Thus, it is difficult to estimate the stiffness of a diffuse lesion using strain imaging because the technology cannot really estimate minor differences in stiffness. By contrast, SWE uses a push pulse, known as the ARFI, which generates a shear wave in a focused area within the ROI [1]. We surmise that the SWV only reflects the characteristics of the tissue inside the ROI; thus SWE, but not strain elastography, may be able to diagnose diffuse lesions such as CAT. In other words, SWE can examine the pathological structures within the target region inside the ROI.

Diagnostic criteria for CAT used in the present study included low echogenicity on ultrasonography. Previous studies have shown that the duration of inflammation correlates with the degree of sonographic hypoechogenicity [15, 20]. Thus, it is plausible that CAT associated with hypoechogenicity on ultrasonography reflects the presence of fibrosis in the present study. These results indicate that a combination of B-mode scanning and SWE could be used to evaluate the degree of fibrosis in CAT.

SWV in lobes with CAT was positively correlated with the thickness of the thyroid isthmus. Since CAT is an inflammatory disease, the duration of disease affects the progression of fibrosis and the size of diffuse lesions [15, 16]. We think that this explains the positive correlation between SWV and the thickness of the thyroid isthmus.

The presence of anti-thyroperoxidase antibodies may be characteristic of a late adaptive immune response, whereas anti-thyroglobulin antibodies may reflect an early immune response [16, 20]. Anti-thyroperoxidase antibodies are closely associated with overt thyroid dysfunction, and their presence tends to correlate with the degree of thyroid damage and lymphocytic infiltration [16]. Therefore, anti-thyroperoxidase antibody titers may correlate with the degree of fibrosis in the thyroid, which may explain the observed positive correlation between SWV and serum titers of anti-thyroperoxidase antibodies. On the other hand, anti-thyroglobulin antibodies are thought to reflect an early immune response and the relationship between anti-thyroglobulin antibodies and the duration of disease is unclear. Fibrosis does not progress during the early stages of the immune response, which may explain why serum titers of anti-thyroglobulin antibodies were not correlated with SWV.

Previous studies have reported that some thyroid nodules are unmeasurable [1, 4, 21–23]. However, we were able to quantitatively measure all normal and CAT thyroids in the present study. Some authors have reported that tumors with heterogeneous histopathology within a given ROI were unmeasurable, because SWV cannot be calculated when the SWV varies within the ROI. It is well known that some types of papillary thyroid carcinoma, which are composed of solid cells, fibrosis, adipose tissue, and other components, are unmeasurable [24]. This study shows that quantitative SWE is useful for making measurements in diffuse targets stable.

A limitation of this study was that pathological examination results are not available for all patients.

## 5. Conclusions

Quantitative SWE but not strain elastography is useful for diagnosing CAT. SWE can also be used to evaluate the degree of fibrosis in CAT.
